# Decease of Prof. Horner

**Published:** 1853-04

**Authors:** 


*Decease of Prof. Horner.* — We are pained to announce the death of the distinguished anatomist, Dr. Wm. E. Horner. He died of disease of heart. Dr. Horner held the chair of anatomy in the University of Pennsylvania, for nearly, if not quite a quarter of a century. His works on anatomy have long been recognized as standard text books in that department of science. In his early professional life he was connected with the army, and served, during the last war with Great Britain, on the Niagara frontier. Some interesting reminiscences of this period of army service, from his pen, have lately appeared in the Philadelphia Medical Examiner. In his decease science looses one of its most distinguished votaries; but his justly earned reputation, and many excellencies, still remain to confer honor on the American medical profession.

